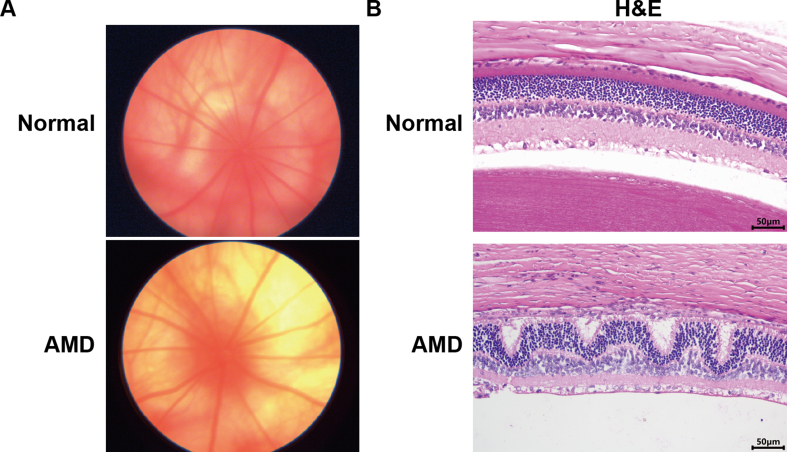# Investigation of the temporal distribution of anti-VEGF drugs in the retina and the correlation with the distribution of FcR isoforms

**DOI:** 10.1016/j.gendis.2025.101698

**Published:** 2025-05-28

**Authors:** Jicai He, Yanping Jiang, Rongqin Yang, Ziwen Lu, Zhihuan Li, Zhigang Tu

**Affiliations:** a Department of Ophthalmology, The First People's Hospital of Liangshan, Xichang, Sichuan 615000, China; b Xichang Medical College, Xichang, Sichuan 615013, China; c School of Life Sciences, Jiangsu University, Zhenjiang, Jiangsu 212013, China

Neovascular age-related macular degeneration (nAMD) and diabetic macular edema are leading causes of vision loss in aging populations, correlating closely with elevated vascular endothelial growth factor (VEGF) levels that raise the permeability of capillaries, leading to the disruption of the blood-retinal barrier and detachment of the retinal neuroepithelial layer. The profound success of anti-VEGF treatments has spurred extensive pharmaceutical research and development.

Anti-VEGF drugs for such retinal conditions can be categorized into larger molecules with Fc fragments (*e.g.*, bevacizumab) and smaller molecules without Fc fragments (*e.g.*, ranibizumab and brolucizumab). Fc receptors, prevalent in ocular tissues, allow anti-VEGF drugs with Fc fragments to bind to retinal pigment epithelial cells, impacting distribution and metabolism. Drugs without Fc fragments, such as ranibizumab and brolucizumab, easily reach the deep retina through diffusion, while transportation of those with Fc fragments, like bevacizumab, is more likely influenced by Fc receptors.^1^ For instance, ranibizumab penetrates all retinal layers,^2^ while only 2% of aflibercept, which contains Fc fragments, reaches the retina-choroid.^3^ Accordingly, ranibizumab relieved retinal edema within 7 days, whereas aflibercept takes approximately 4 weeks.^4^ Currently, there is a significant gap in experimental evidence concerning the transportation and distribution of anti-VEGF drugs with or without Fc fragments within the retina.

Bevacizumab, a humanized full-length anti-VEGF monoclonal antibody with Fc fragments, is widely used off-label. Ranibizumab, an affinity-matured and antigen-binding fragment without Fc, targets VEGF-A for treating nAMD and diabetic macular edema.^2^ Brolucizumab, a low-molecular-weight VEGF inhibitor without Fc fragments, is a single-chain antibody fragment designed for nAMD, diabetic macular edema, and macular edema following retinal vein occlusion.

In our studies on normal rats, intravitreal injections of brolucizumab, ranibizumab, and bevacizumab showed no adverse effects like inflammation, confirming their safety. We assessed the penetration of these drugs into the retina using ELISA assays and 3D computational models that subdivided the rat retina into Inner, Outer, and Deep layers. The Inner layer lies between the internal limiting membrane and inner nuclear layer without their edges; the Outer layer is in fact the outer plexiform layer; the Deep layer is the layer of rods and cones. Following intravitreal injection, all three drugs rapidly penetrated the vitreous body and reached peak concentrations in the retina within 6 h. Brolucizumab and ranibizumab levels then gradually declined, while bevacizumab concentrations significantly dropped within 24 h (Fig. 1A). Immunohistochemistry assays further demonstrated that they rapidly reached peak concentrations in the Inner layer at 6 h (Fig. 1B–G), indicating effective penetration without significant barriers.

**Figure 1:**
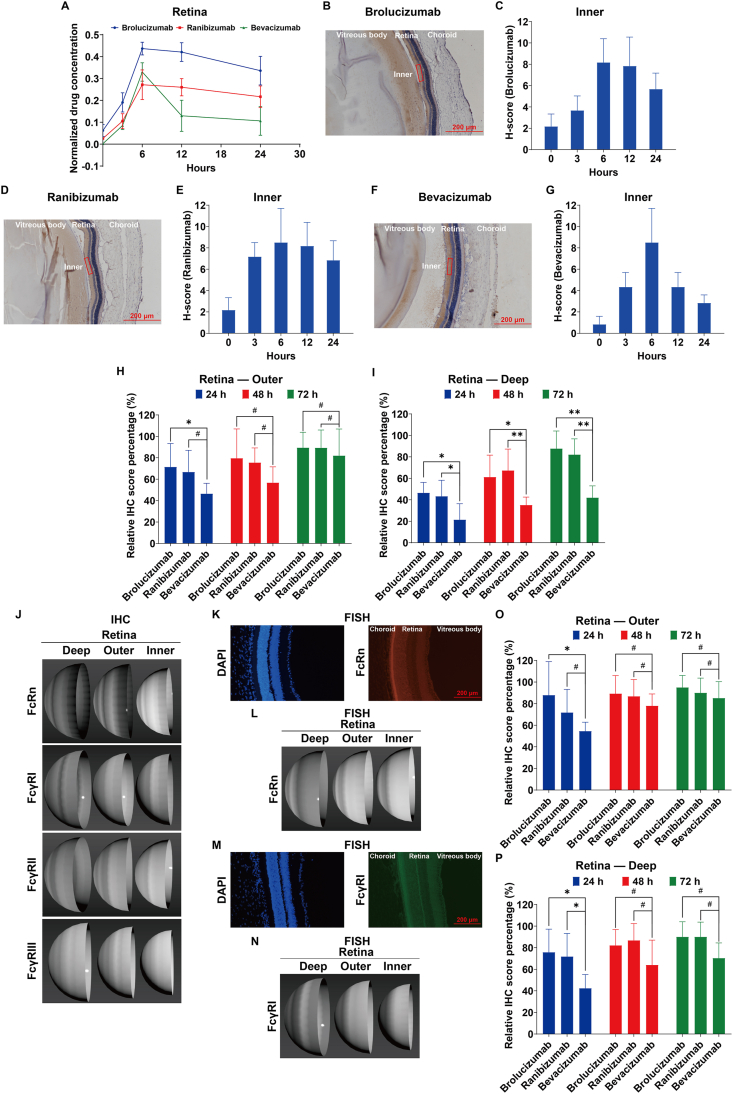
Distribution of brolucizumab, ranibizumab, and bevacizumab in the retina of normal rat eyes and of rat eyes in age-related macular degeneration (AMD) conditions, and distribution of FcR isoforms in the retina. **(A)** ELISA detection of the distribution of brolucizumab, ranibizumab, and bevacizumab in the retina of normal rat eyes. **(B, C)** Immunohistochemical detection of brolucizumab in normal rat eye. Representative images (B) and immunohistochemical scoring statistics of the area between the internal limiting membrane and the inner nuclear layer (Inner) (C) are shown. **(D, E)** Immunohistochemical detection of ranibizumab diffusion in normal rat eyeballs. Representative images (D) and immunohistochemical scoring statistics of the area between the internal limiting membrane and the inner nuclear layer (Inner) (E) are shown. **(F, G)** Immunohistochemical detection of bevacizumab diffusion in normal rat eyeballs. Representative images (F) and immunohistochemical scoring statistics of the area between the internal limiting membrane and the inner nuclear layer (Inner) (G) are shown. Data shown are mean ± standard deviation of animal data (*n* = 5) in each experimental group, unless otherwise specified. **(H)** Distribution of brolucizumab, ranibizumab, and bevacizumab in the Outer plexiform layer (Outer) between the Outer nuclear layer and the Inner nuclear layer of the retina of normal rat eyes at different time points. **(I)** Distribution of brolucizumab, ranibizumab, and bevacizumab in the retinal rods and cones area (Deep) of normal rat eyes at different time points. Data shown are mean ± standard deviation of animal data (*n* = 5) in each experimental group, unless otherwise specified. Statistically significant differences with *p* < 0.05 were considered significant (^#^*p* > 0.05, ∗*p* < 0.05, and ∗∗*p* < 0.01). **(J)** Immunohistochemistry experiments were conducted to assess the expression of four FcR subtypes (FcRn, FcγRI, FcγRII, and FcγRIII) proteins in the normal rat eyeball retina. Using 3DS MAX, we created 3D models to visualize protein expression scores in different structural areas of the retina, mainly Inner, Outer, and Deep. **(K–N)** Fluorescence *in situ* hybridization experiments revealed the expression of two FcR subtypes (FcRn, FcγRI) mRNA in the normal rat eyeball retina. Representative images for FcRn (K) and FcγRI (M) were selected. Using 3DS MAX, we also created 3D models to visualize mRNA expression scores in different structural areas of the retina, mainly Inner, Outer, and Deep, respectively (L, N). **(O)** Distribution of brolucizumab, ranibizumab, and bevacizumab in the Outer plexiform layer (Outer) between the Outer nuclear layer and the inner nuclear layer of the retina of AMD rat eyes at different time points. **(P)** Distribution of brolucizumab, ranibizumab, and bevacizumab in the retinal rods and cones area (Deep) of AMD rat eyes at different time points. Data shown are mean ± standard deviation of animal data (*n* = 5) in each experimental group, unless otherwise specified. Statistically significant differences with *p* < 0.05 were considered significant (^#^*p* > 0.05 and ∗*p* < 0.05).

Later, we examined the penetration dynamics in the Outer and Deep layers. At 24 h post-intravitreal injection, brolucizumab showed 70 % penetration in the Outer layer, compared with bevacizumab's 45% when normalized to the Inner layer H-scores at 6 h (Fig. 1H). Over time, levels of all drugs in the Outer layer increased similarly at 48 h and 72 h. In the Deep layer, brolucizumab and ranibizumab levels were significantly higher than bevacizumab's at 24 h, 48 h, and 72 h, indicating better access to the Deep layer within 72 h post-intravitreal injection (Fig. 1I).

Previous studies have indicated that Fc fragments play key roles in antibody uptake, metabolism, and distribution. The major receptors for these Fc-fragment containing antibodies are Fc-receptors, including Fcγ-receptors (FcγR) I, FcγRII, and FcγRIII on the cell membrane and the neonatal Fc-receptor (FcRn) expressed on the cell membrane and intracellularly for IgG. Different FcRs have varying affinities for antibodies, so mapping their distribution in the retina may help understand ophthalmic medications and support the development of novel drugs. All these FcRs were detected in normal rat retinas via immunohistochemistry assays (Fig. 1J). 3D mappings of FcRs in the Deep, Outer, and Inner layers showed the highest protein levels in the Deep layer and the lowest in the Inner layer. Data suggest that FcRn and FcγRI are the major isoforms in normal rat retinas. mRNA distribution for FcRn (Fig. 1K, L) and FcγRI (Fig. 1M, N), examined by fluorescent *in situ* hybridization, presented a similar expression pattern.

Since the retinal layers' anatomy can be altered due to AMD, the distribution of brolucizumab, ranibizumab, and bevacizumab was reassessed via immunohistochemistry assays in AMD models (Fig. S1). The data demonstrated that 24 h post-intravitreal injection, brolucizumab levels in the Outer layer were significantly higher than bevacizumab when normalized to the Inner layer H-scores at 6 h (Fig. 1O). However, by 48 h and 72 h, the distribution of all three drugs in the Outer layer evened out with no significant differences observed.

Meanwhile, brolucizumab and ranibizumab showed significantly higher expression in the Deep retinal layer 24 h post-intravitreal injection compared with bevacizumab in AMD models (Fig. 1P). However, bevacizumab levels in the Deep layer increased rapidly, equalizing the distribution among all three by 48 h and 72 h post-intravitreal injection. This suggests that AMD-related structural changes enhance retinal permeability, allowing accelerated entry of bevacizumab into both the Outer and Deep layers. Yet, within 24 h, bevacizumab's permeability remains notably lower than that of brolucizumab and ranibizumab.

Research indicates that the diffusion rates of antibodies to the retina correlate inversely with their size. Bevacizumab is therefore more difficult to diffuse through the dense, multilayered structure of the retina into the Deep layers due to its size. Ranibizumab diffuses passively to the Deep layers through the intercellular space,^2^ whereas Fc-containing antibodies rely on active transport via FcRs.

FcRs in the retina bind to molecules containing Fc, allowing them to enter cells, degrade some portions, and re-transport a small portion out of the cells. Therefore, the retina exhibits a retention effect on drugs with Fc. Our data and those of the other studies suggest that compared with brolucizumab and ranibizumab, bevacizumab's penetration capability appears limited, potentially due to its Fc fragments. Our results and several other studies demonstrate that ranibizumab and brolucizumab are more likely to penetrate deeply into the retina and even reach the choroid,^2^ acting in areas that are difficult to access for Fc fragment-containing drugs. These findings align with the rapid therapeutic response of these drugs observed in clinical settings and suggest that ranibizumab and brolucizumab may be superior options for treating conditions such as deep retinal or choroidal edema and choroidal neovascularization, as supported by clinical outcomes.^5^ Additionally, brolucizumab had a significant impact on choroidal morphology in patients with type 1 nAMD compared with aflibercept.

Despite its generally low transport efficiency, bevacizumab can still reach the Deep retinal layers in eyes affected by AMD, likely because the disease could increase intercellular spaces and disrupt the structure of the retina.

This study indicates that brolucizumab and ranibizumab quickly penetrate the Deep retinal layers in healthy rats; while all three drugs achieve Deep retinal penetration in AMD-affected rats, with brolucizumab and ranibizumab showing faster penetration. These findings align with the quick therapeutic response of ranibizumab seen in clinics and indicate that ranibizumab and brolucizumab may be better options for treating conditions like deep retinal or choroidal edema and choroidal neovascularization, corroborating clinical study outcomes.

## CRediT authorship contribution statement

**Jicai He:** Conceptualization, Formal analysis, Investigation, Supervision, Writing – review & editing, Writing – original draft. **Yanping Jiang:** Formal analysis, Investigation, Writing – review & editing. **Rongqin Yang:** Formal analysis, Investigation, Writing – review & editing. **Ziwen Lu:** Formal analysis, Investigation, Writing – review & editing. **Zhihuan Li:** Formal analysis, Investigation, Writing – review & editing. **Zhigang Tu:** Conceptualization, Writing – original draft, Writing – review & editing.

## Ethics declaration

All procedures involving rats were approved by the Animal Care and Use Committee of Jiangsu University and followed the standard in the ARVO Animal Statement for the Use of Animals in Ophthalmic and Vision Research and Animal Research.

## Funding

This study was supported by the Investigator Initiated Research Fund from Beijing Novartis Pharma Co., Ltd. (to Z.T.).

## Conflict of interests

Z.T. reports financial support provided by Beijing Novartis Pharma Co., Ltd. There are no additional relationships, no patents, and no additional activities to disclose.

## Figures and Tables

**figs1:**