# Supplementary material for: Investigation of the temporal distribution of anti-VEGF drugs in the retina and the correlation with the distribution of FcR isoforms
Source: Genes Dis. 2025 May 28;12(6):101698. doi: 10.1016/j.gendis.2025.101698 (PMC12343353; doi:10.1016/j.gendis.2025.101698)
Supplement: Multimedia component 1 [file mmc1.doc]

Supplementary Methods

Reagents and antibodies

The FcRn-specific polyclonal antibody (FcRn), polyclonal FCGR2A/CD32a antibody (FcγRII), and polyclonal CD16 antibody (FcγRIII) were purchased from Proteintech (Wuhan, Hubei, China). Polyclonal CD64 antibody (FcγRI) and goat anti-human IgG Fc (HRP) were purchased from Abcam (Waltham, MA, USA). Goat anti-human IgG Fc secondary antibody (HRP) and goat anti-human IgG Fc cross-absorbed secondary antibody (DyLight 594) were purchased from Thermo Fisher Scientific (Waltham, MA, USA). Peroxidase affinipure mouse anti-human IgG F(ab')_2_ fragment was purchased from Jackson ImmunoResearch (West Grove, PA, USA). The adhesive slides and coverslips were purchased from Citotest Scientific, (Nanjing, Jiangsu, China). Chemical reagents were purchased from Sinopharm (Shanghai, China) unless otherwise specified. Tissue-Tek O.C.T. Compound was purchased from SAKURA Finetek (Torrance, CA, USA). Ranibizumab was obtained from Hongji Biotech (Shenzhen, Guangdong, China).

Animal models

All procedures involving rats were approved by the Animal Care and Use Committee of Jiangsu University and followed the standard in the ARVO Animal Statement for the Use of Animals in Ophthalmic and Vision Research and Animal Research. Normal male Sprague-Dawley (SD) rats weighing 180 to 200 g were purchased from Cavins Laboratory Animal Co. (Changzhou, Jiangsu, China). AMD rat models weighing about 200 g were purchased from ZHBY Biotech (Nanchang, Jiangxi, China). Rats are frequently used in ophthalmic medicine studies and for creating AMD models, as their eyeball sizes are well-suited for such experiments [1-3]. The animals were all raised in the Experimental Animal Center of Jiangsu University and provided with sterilized normal food and water and housed in a barrier facility with the temperature at 23±3°C and the humidity at 55±15% under a 12-hour light-dark cycle, where they had free access to food and water. The rats were anesthetized using small animal anesthesia machine with isoflurane (RWD Life Science, Shenzhen, Guangdong, China). In the experiments, the HAMILTON microsyringes with 33G needles were carefully inserted into the vitreous bodies of the rats’ eyeballs from the corneal edge. The dosages for animal experiments were converted for rats based on the clinical dosages of the drugs. The rats were randomized into four groups (vehicle groups, 0.108 mg/200 g brolucizumab, 0.009 mg/200 g ranibizumab, and 0.0225 mg/200 g bevacizumab). The sample sizes for each group were determined based on previous studies [2, 4-7]. 5 μL of drugs were gently injected into the vitreous bodies. For the control group, an equal volume of normal saline (5 μL) was also gently injected into the vitreous bodies. Following injections, the needles were withdrawn after the specified retention periods. The rats were kept under regular conditions, and the specimens were collected at the following time points: 0, 3, 6, 12, 24, 48, and 72 hours (5 rats/time point). At the end of the observation, rats were sacrificed and autopsied.

Tissue preparation

Following anesthesia, the eyes were enucleated, and then fixed with Davidson’s fixative (neutral formaldehyde, 95% ethanol, glacial acetic acid, and distilled water in a ratio of 2:3:1:3) for IHC assays or promptly frozen in OCT embedding matrix for fluorescence in situ hybridization (FISH) assays. For the preparation of fresh specimens of the retina tissues for enzyme-linked immunosorbent assay (ELISA) assays, the eye globes were quickly frozen with liquid nitrogen and dissected using a dissecting microscope (NOVEL BM2000, Nanjing, Jiangsu, China). Sections were cut at a thickness at 5 µm for paraffin sections and 10 µm for frozen sections from the ciliary bodies to the optic nerves.

Immunohistochemistry (IHC) assays

The IHC assays were performed as previously described [8]. In summary, paraffin sections underwent a series of treatments including drying, dewaxing, and rehydration. For paraffin sectioning, the eyeball tissues were fixed in Davidson’s fixative for 24 hours. After fixation, the eyeballs underwent gradual dehydration using alcohol with increasing concentrations (45%, 55%, 65%, 75%, 85%, 95%, and absolute ethanol) for 1 hour per concentration. The dehydrated eyeballs were then immersed in paraffin at 58-60°C for 3 hours and embedded in the corresponding paraffin blocks. Antigen retrieval was performed through high-temperature, high-pressure treatment in citrate buffer solution (pH 6.0). Then sections were treated with a 3% H_2_O_2_ solution to block endogenous catalase activity. Subsequently, the tissue sections were incubated with primary antibodies overnight in a humidified chamber at 4℃. After that, sections were treated with the secondary antibody for 20 minutes and 3,3'-diaminobenzidine (DAB) for 3-5 minutes. Immunostained sections were lightly counterstained with hematoxylin for observation and imaging. Citrate buffer solution, 3,3'-diaminobenzidine (DAB), and the hematoxylin staining kit were all purchased from Gene Tech (Shanghai, China). The images were captured using the Nikon Eclipse fluorescence microscope (Tokyo, Japan).

Immunohistochemical assessments were independently scored by two blinded investigators. The overall scores were determined by multiplying the scores for staining intensity and stained areas of the sections. The staining intensities were graded as 0 (negative), 1 (weak), 2 (moderate), and 3 (strong). The stained areas were scored as 0 (0%), 1 (1-25%), 2 (26-50%), 3 (51-75%), and 4 (76-100%), based on the proportion of the positive cells. In cases of significant disparity between the two scores, the slides were reassessed.

FISH assays

The experimental procedure followed the guidelines provided in the Fluorescence In Situ Hybridization (FISH) kit (Genepharma, Suzhou, Jiangsu, China). In summary, tissue sections were then treated with proteinase K, denatured, subjected to hybridization, and stained for nuclei. The specimens were then sealed and examined under the Nikon Eclipse fluorescence microscope (Tokyo, Japan), with images captured for further analysis.

ELISA assays

In such assays, a standard gradient dilution solution of the protein was applied to coat a 96-well plate. Simultaneously, the test samples were also diluted incrementally and added to the plate for coating. The plate was then incubated overnight at 4°C. After incubation, a 0.5% BSA solution was used to block the wells at 37°C for 1 hour. Subsequently, the plates were incubated with the antibody, and excess antibody-HRP diluent was added to the wells, followed by another incubation at 37°C for 1 hour. For the color reaction, a chromogenic solution was introduced into the wells. After the color had fully developed, a 2M H_2_SO_4_ solution was used to halt the reaction. Absorbance readings were taken at 450 nm using a microplate reader (DeTie HBS-1101, Nanjing, Jiangsu, China).

Construction of three-dimensional (3D) computational retina model

3D computational retina models were constructed to mimic the retina anatomy of rat eyes using 3ds MAX (Autodesk, San Rafael, California, USA; version 2022.24.0.0.923). The leftmost top of the hemisphere in the model corresponds to the posterior portion of the eyeball, pointing to the optic nerve. In our experiments, the retina was roughly divided into three layers: the Inner, Outer, and Deep layers. The Inner layer lies between the internal limiting membrane and inner nuclear layer without the their edges; the Outer layer is in fact outer plexiform layer; and the Deep layer is the layer of rods and cones. In this model, grayscales were determined by scoring the stained levels in the images from FISH and IHC assays via Adobe Photoshop CS6 (San Jose, California, USA; version 13.0), indicating the expression of the proteins or mRNAs detected. Ten cross section loci evenly located on the retina which are perpendicular to the optic nerves at the same intervals were used to build 3D retina models for either IHC or FISH assays and six sections were used for each locus. For FISH image analysis, the values of the stained areas in the G/R channel in the RGB color format were denoted as A, representing the fluorescence intensities; the values of the background area of the G channel were denoted as B, representing the background brightness. Similarly, for IHC image analysis, the values of the stained areas in the R+G channel were denoted as A, representing the staining intensities; the values of the background area in the R+G channel were denoted as B, representing the background. Averages of these values were all calculated in five different fields. Then the staining intensities of FISH and IHC assays were calculated as X=|a-b|, representing the relative intensities of staining, where X_min_ and X_max_ are the lowest and highest X values in a group, respectively. Thus, the grayscale G=(X-X_min_)/(X_max_-X_min_)*100%. When G is 100, it is black in the model, indicating the highest expression of protein or mRNA. When Y is 0, the result is white, indicating the lowest expression of protein or mRNA.

Statistical analysis

All Data are reported as mean ± SD, unless otherwise stated. Statistically significant differences were analyzed by using one-way ANOVA with GraphPad Prism version 9.00 (GraphPad, San Diego, CA, USA), with P < 0.05 considered significant (* P < 0.05; ** P < 0.01; *** P < 0.001) unless otherwise specified.

References

1. Goo, H., et al., *Multi-Wavelength Photobiomodulation Ameliorates Sodium Iodate-Induced Age-Related Macular Degeneration in Rats.* Int J Mol Sci, 2023. 24(24).

2. Koster, C., et al., *Sodium-Iodate Injection Can Replicate Retinal Degenerative Disease Stages in Pigmented Mice and Rats: Non-Invasive Follow-Up Using OCT and ERG.* Int J Mol Sci, 2022. 23(6).

3. Grossniklaus, H.E., S.J. Kang, and L. Berglin, *Animal models of choroidal and retinal neovascularization.* Prog Retin Eye Res, 2010. 29(6): p. 500-19.

4. de Cogan, F., et al., *Topical Delivery of Anti-VEGF Drugs to the Ocular Posterior Segment Using Cell-Penetrating Peptides.* Invest Ophthalmol Vis Sci, 2017. 58(5): p. 2578-2590.

5. Horita, S., et al., *Species differences in ocular pharmacokinetics and pharmacological activities of regorafenib and pazopanib eye-drops among rats, rabbits and monkeys.* Pharmacol Res Perspect, 2019. 7(6): p. e00545.

6. Choi, E.J., et al., *A Novel Eye Drop Candidate for Age-Related Macular Degeneration Treatment: Studies on its Pharmacokinetics and Distribution in Rats and Rabbits.* Molecules, 2020. 25(3).

7. Shen, G., et al., *Kallistatin Deficiency Induces the Oxidative Stress-Related Epithelial-Mesenchymal Transition of Retinal Pigment Epithelial Cells: A Novel Protagonist in Age-Related Macular Degeneration.* Invest Ophthalmol Vis Sci, 2023. 64(12): p. 15.

8. Lu, Z., et al., *Partitioning defective 6 homolog alpha (PARD6A) promotes epithelial-mesenchymal transition via integrin beta1-ILK-SNAIL1 pathway in ovarian cancer.* Cell Death Dis, 2022. 13(4): p. 304.
