# Supplementary material for: Investigation of the temporal distribution of anti-VEGF drugs in the retina and the correlation with the distribution of FcR isoforms
Source: Genes Dis. 2025 May 28;12(6):101698. doi: 10.1016/j.gendis.2025.101698 (PMC12343353; doi:10.1016/j.gendis.2025.101698)
Supplement: Multimedia component 2 [file mmc2.doc]

**Supplementary Figure legend**

**Figure S1. Successful Construction of AMD models of rats. A.** The fundus conditions of normal rats and AMD models of rats were examined using color fundus photography (CFP). **B.** Representative H&E staining images of eyes of normal rats and AMD models. The retina of normal rats had normal morphology, clear structural layers, uniform coloration, regular outer nuclear layers, and no obvious pathological changes. The structure of the AMD model was disordered, and the inner and outer nuclear layers of the retina were arranged disorderly. The boundaries were unclear, and the outer nuclear layer appeared wavy.
